# Dimerization and thiol sensitivity of the salicylic acid binding thimet oligopeptidases TOP1 and TOP2 define their functions in *redox*-sensitive cellular pathways

**DOI:** 10.3389/fpls.2015.00327

**Published:** 2015-05-18

**Authors:** Timothy J. Westlake, William A. Ricci, George V. Popescu, Sorina C. Popescu

**Affiliations:** ^1^The Boyce Thompson Institute for Plant ResearchIthaca, NY, USA; ^2^Department of Plant Pathology and Plant Microbe Biology, Cornell UniversityIthaca, NY, USA; ^3^Department of Biology, West Chester University of PennsylvaniaWest Chester, PA, USA; ^4^National Institute for Lasers, Plasma and Radiation PhysicsBucharest, Romania

**Keywords:** thimet oligopeptidase, salicylic acid, redox potential, systems model, oxidative stress

## Abstract

A long-term goal in plant research is to understand how plants integrate signals from multiple environmental stressors. The importance of salicylic acid (SA) in plant response to biotic and abiotic stress is known, yet the molecular details of the SA-mediated pathways are insufficiently understood. Our recent work identified the peptidases *TOP1* and *TOP2* as critical components in plant response to pathogens and programmed cell death (PCD). In this study, we investigated the characteristics of *TOPs* related to the regulation of their enzymatic activity and function in oxidative stress response. We determined that TOP1 and TOP2 interact with themselves and each other and their ability to associate in dimers is influenced by SA and the thiol-based reductant DTT. Biochemical characterization of TOP1 and TOP2 indicated distinct sensitivities to DTT and similarly robust activity under a range of pH values. Treatments of *top* mutants with Methyl Viologen (MV) revealed *TOP1* and *TOP2* as a modulators of the plant tolerance to MV, and that exogenous SA alleviates the toxicity of MV in *top* background. Finally, we generated a TOP-centered computational model of a plant cell whose simulation outputs replicate experimental findings and predict novel functions of TOP1 and TOP2. Altogether, our work indicates that *TOP1* and *TOP2* mediate plant responses to oxidative stress through spatially separated pathways and positions proteolysis in a network for plant response to diverse stressors.

## Introduction

Plants are dynamic living systems wherein external and internal signals induce changes over time. Plant cells decode signals from varied and often concurrent stressors in order to mount appropriate defenses. Salicylic acid (SA) is a small phenolic molecule with hormonal properties that plays critical roles in plant stress response to biotic and abiotic factors (Rivas-San Vicente and Plasencia, 2011; Denancé et al., 2013). The discovery of SA-binding proteins revealed that SA-mediated signaling and perception involves interactions of SA with multiple protein partners (An and Mou, 2011; Moreau et al., 2013). The apparent complexity of SA-mediated immune pathways in regards to the number and regulatory mechanisms of participating components, is evidenced by the diversity of cellular and plant-level physiological outcomes that include oxidative bursts, programmed cell death (PCD), and local and systemic pathogen resistance. Therefore, integrative approaches that merge experimental and analytical approaches applied to the study of SA cellular pathways would be invaluable in uncovering both the mechanistic details of cellular elements or processes under SA regulation and the general rules that govern the functioning of immune pathways and plants response to environment.

Previously, we used a protein microarray screen to identify two SA-binding proteins—the thimet oligopeptidases TOP1 and TOP2 classified in as putative zinc- and thiol-dependent endopeptidases based on homology to the metazoan counterpart (Moreau et al., 2013). Biochemical assays verified that both TOP1 and TOP2 bind SA with distinct affinities and revealed that SA inhibits non-competitively TOP1 and TOP2's peptidase activities (Moreau et al., 2013). *In vitro* biochemical evidence suggests that TOP1 is a component of organellar proteolytic machinery; TOP1 is predicted to participate in the degradation of imported proteins' signal sequences and potentially to play a broader role in general organellar peptide degradation (Kmiec et al., 2013; Moreau et al., 2013). Conversely, *TOP2* encodes a cytosolic peptidase; biochemical evidence implicates TOP2 in the proteolytic machinery downstream of the 26S proteasome and TOP2 was hypothesized to prevent the accumulation of free peptides generated from oxidative stress (Polge et al., 2009). The potential functions of plant TOPs in the regulated proteolysis mirrors those of the metazoan TOP which plays active roles in controlling the accumulation of bioactive neuropeptides, hormones, and antigenic peptides (Chu and Orlowski, 1985; York et al., 2003; Shivakumar et al., 2005). Our previous work determined that *TOP1* and *TOP2* are components of the immune response (Moreau et al., 2013). Altered expression of *TOPs* inhibited plant response to pathogens that induce effector-triggered immunity and the development of pathogen-triggered PCD. Further exploration into stress-related functions of *TOPs* established that *TOP1* and *TOP2* are necessary for plant response to high concentrations of exogenous SA (Moreau et al., 2013), and brought forth new questions about TOPs enzymatic characteristics and their specific roles in the oxidative stress response.

Controlled oxidative bursts—characterized by rapid accumulation of reactive oxygen species (ROS) in the apoplast, cytosol, and organelles—are a common characteristic of cellular stress caused by biotic and some abiotic factors (Wrzaczek et al., 2013). In both dicots and monocots, SA influences the accumulation of ROS during stress response and consequently, cell survival. SA-mediated oxidative and reductive bursts can lead to *redox*-based modifications of sensors which are proteins with higher chemical reactivity whose location and ionic state render them sensitive to oxidation by ROS (Mou et al., 2003). Interestingly, metazoan TOPs are described as thiol-dependent peptidases since their activity is markedly altered by thiols such as dithiothreitol (DTT) (Tisljar and Barrett, 1990) and are considered as likely participants in the cellular *redox* reactions where thiols are part of the antioxidant defense and signaling processes (Ferreira et al., 2013). Currently, the identity of plant *redox* sensors and the mechanistic basis of the complex relationship that exists between SA and ROS homeostasis remain largely unknown (Foyer and Noctor, 2013).

To understand how multiple cellular components cooperate and influence each other to generate appropriate physiological outputs to pathogen infection or environmental stress, it is critical to establish a platform for the system-level study of plant stress response. Computational models that represent the known structure and dynamics of stress-related pathways could uncover novel relationships and facilitate a predictive understanding of plants at molecular level. The complementarity of *TOP1*-/*TOP2*-mediated stress responses, their distinct spatial localization, levels of expression, and enzymatic activity, makes the computational study of their system-level dynamics necessary and potentially insightful for unraveling the complexity of SA pathways.

In this study, we describe novel functions of *TOP1* and *TOP2* and characterize aspects of TOP peptidases that may relate to their cellular regulation by SA and the reduction-oxidation cellular environment in the context of plant response to oxidative stress.

## Results

### TOP1 and TOP2 form dimers in plant cells

**Figure 1:**
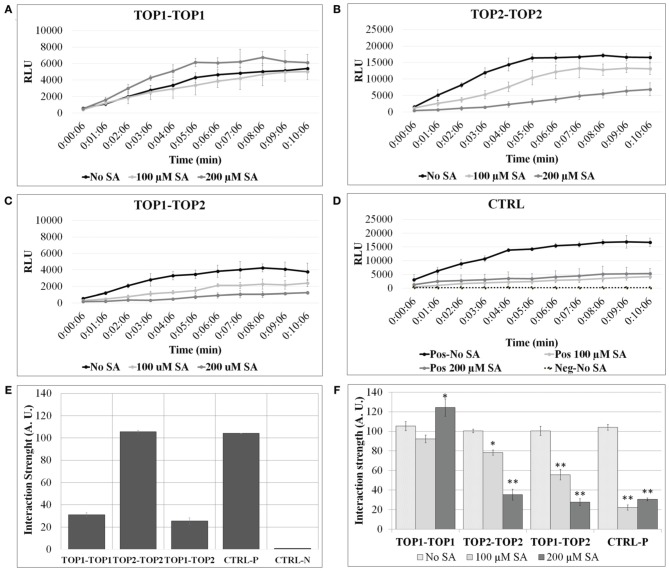
**TOP1 and TOP2 dimerize in a SA concentration-dependent manner**. Split-luciferase assays were performed in *Arabidopsis thaliana* protoplasts in the presence or absence of SA. Detection of relative luminesce units (RFU) emitted was used to assess potential binding of the interacting pairs. **(A)** The relative luminescence of TOP1-TOP1 interactions over time in the presence or absence of SA. **(B)** The relative luminescence of TOP2-TOP2 interactions over time in the presence or absence of SA. **(C)** The relative luminescence of TOP1-TOP2 interactions in the presence or absence of SA. **(D)** The relative luminescence of control interacting partners (MKK5 and HopF2) in the presence or absence of SA. **(E)** TOP1 and TOP2 form homo- and hetero-dimers *in vivo*. Bars represent the normalized interaction strength, measured as *Renilla* luciferase intensity values normalized to a positive interaction set of proteins (Ctrl-P). **(F)** The interactions between TOP1 and TOP2 are modulated by salicylic acid (SA). Bars show interaction strength in SA and non-SA conditions. For both (**A,B)**, the luciferase intensity values measured in the 07:06 and 10:06 min time interval after adding the luciferase substrate, were used to calculate the relative interaction strength of the TOP interactions (A.U. are arbitrary units); all intensity values were normalized to the values at 07:06 min time point for the CTRL-P protein pair and averaged for plotting; the error bars are standard deviations calculated relative to the negative control (CTRL-N) in **(A)**, and relative to the “no-SA” condition for each protein pair tested in **(B)**. Asterisks represent statistical significance (Student's *T*-test) (^*^p < 0.05 and ^**^p < 0.01) calculated from 3 to 6 replicates per protein pair tested.

We examined the propensity of TOP1 and TOP2 to assemble in homo- and hetero-dimers using two distinct methods. First, we utilized a split-luciferase complementation assay (SLCA) that allowed for observation of protein-protein interactions within the context of the plant cell environment (Fujikawa and Kato, 2007). *Arabidopsis* protoplasts were prepared and co-transformed with pairs of plasmid constructs containing the coding sequences of *TOP1* or *TOP2*, cloned in frame with either the N-terminal or the C-terminal halves of the *Renilla* luciferase coding sequence (TOP1- or TOP2-NLuc and TOP1- or TOP2-CLuc). Interactions between TOPs-Luc fusions were detected by measuring luminescence released upon the restoration of luciferase enzymatic activity. Protoplasts expressing luciferase terminal fusions with known interactors (MKK5 and HOPF2) (Wang et al., 2010), constituted the positive interaction control, while protoplasts expressing TOP1-Luc or TOP2-Luc in pairs with non-interacting proteins constituted the negative controls (Figures 1A–D). We found that the protoplasts expressing various combinations of TOP1-Luc and TOP2-Luc fusions exhibited significantly greater luminescence intensities than those of the negative control; furthermore, the luminescence intensity of the TOP2-TOP2 interaction was significantly greater (3.4-fold higher in average) than that of TOP1-TOP1 or TOP1-TOP2—both of which showed similar levels of luminescence intensity (Figure 1E). The ability of TOP2 and TOP1 to bind to each other suggests that the binding sites of the two peptidases are conserved. TOP1 and TOP2 are homologs with a high degree of similarity; although their N-termini differ with respect to the transit peptide in TOP1, the overall TOP1-TOP2 similarity is approximately 93%. Despite distinct patterns of subcellular localization, it is possible that TOP1 in transit to the organelles binds to TOP2 and the interaction may have a functional significance. Overall, we conclude that both TOP1 and TOP2 have the capacity to dimerize, albeit with distinct affinities.

### TOP1 and TOP2 form transient monomers and dimers

To further investigate the dimerization potential of TOPs, we used gel filtration chromatography that separates proteins on the basis of mass. We first developed a uniform purification procedure for TOPs using His-tagging; full length *TOP1-His*, and *TOP2-His* were expressed in in the *E. coli* BL21 strain and total lysates were run through a His-tag cobalt column to separate TOP1-His and TOP2-His protein preparation which were then chromatographed through a Superdex 200 gel filtration column.

**Figure 2:**
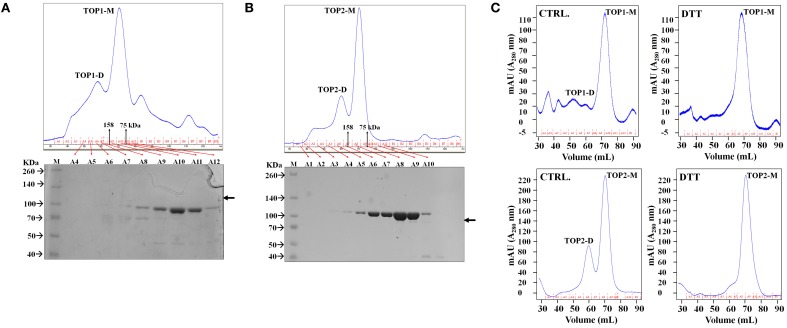
**TOPs Gel Filtration Elution Profile**. Purified bacterially expressed TOP1 and TOP2 are subjected to size exclusion chromatography. **(A)** TOP1 obtained from cobalt His-tagged purification was subjected to size-exclusion chromatography. The elution profile in tandem with SDS PAGE of the fractions indicate that TOP1 elutes primarily at two different sizes corresponding to the sizes of the monomer and dimer. **(B)** Size-exclusion elution profile of TOP2 with corresponding fractions subjected to SDS-PAGE indicate that TOP2 elutes at two peaks corresponding with the dimer and monomer size. **(C)** The elution profile of TOP2 in the absence or after incubation with of dithiothreitol.

Each of the proteins was eluted with two distinct peaks from the gel filtration columns. Protein fractions spanning the elution profiles from both preparations were analyzed by SDS/PAGE and Coomassie staining; TOP1-His and TOP2-His were detected only in the fractions corresponding to the major peaks in the elution profiles (Figures 2A,B). The apparent MW of the elution peaks was estimated by interpolation using the elution profile of conalbumin and aldolase. With the shorter-retention time elution peak, both TOP1-His and TOP2-His eluted at a volumes with approximate MWs 2-fold higher that their actual MWs; by contrast, with the longer retention time peak TOP1-His and TOP2-His eluted at their actual MW. We concluded that the two peaks correspond to the dimeric and monomeric forms, respectively, of TOP1 and TOP2. Based on the total absorption units of each peak, we calculated that the dimer:monomer ratio was approximately 1:3 in the case of each protein, indicating that under the experimental conditions used, the monomeric forms were favored.

Our results indicate that both TOP1 and TOP2 exist as both monomers and dimers; in the case of both TOPs, the monomers are more abundant than dimers in solution.

### SA and dithiothreitol influence the dimer-monomer balance of TOP1 and TOP2

Next, we were interested to investigate a possible functional relevance of the observed TOP dimerization. First, we tested the effect of SA on TOPs homodimerization. SA concentrations and incubation times used in our assays were optimized to maintain protoplast viability. Concentrations of SA beyond 200 μM resulted in significant protoplast lysis during incubation and the lethal effects of SA amounts beyond 500 μM were readily apparent even when the incubation time was shorter. Extending incubation time to over 3 h, in the presence of 100, 200 μM SA or higher SA concentrations, caused significant cell death (data not presented).

To test SA's effects on TOPs interactions, protoplasts expressing various pairs of TOP-Luc fusions were incubated with 100 or 200 μM SA for 3 h and restoration of luciferase activity was measured over time in increments of 1 min. SA treatment significantly lowered the intensity of the reconstituted luciferase in the case of TOP2-TOP2 and TOP1-TOP2 interactions compared to the no-SA condition (Figure 1F). The SA-dependent decrease in the luciferase activity occurred in a concentration dependent manner; 100 μM SA reduced luciferase intensity of TOP2-Luc dimers by approximately 30% and of TOP1-TOP2 dimers by 50%, while 200 μM SA reduced it by 75 and 80%, respectively. On the other hand, SA had a much reduced effect on TOP1-Luc dimerization compared to TOP2-TOP2 and TOP1-TOP2. While 100 μM SA did not significantly impair TOP1 interactions, 200 μM SA slightly increased the luciferase intensity of TOP1-Luc over the no-SA condition within the time interval with the maximum interaction strength. We can't preclude the possibility that the null/low SA sensitivity of the TOP1 dimer in this system is a result of its localization in chloroplasts and mitochondria or that the exogenous SA influences the cytosolic TOP1 in transit to the chloroplasts. The amount of the exogenously applied SA that may be transported into the organelles within our experimental timeframe is unknown; it may be that SA does not accumulate to a threshold high enough to elicit an effect on TOP1 dimerization. The intensity of protoplasts expressing the positive interaction pair was decreased to similar levels (75%) in presence of 100 or 200 μM SA compared to the no-SA control. We conclude that TOP1 *in vivo* dimerization is more resistant to exogenous SA than TOP2 dimerization and both were more resistant than the control interaction pair.

The above results suggest that SA-induced shifts in the reducing-oxidative (*redox*) environment of the protoplasts might interfere with TOP monomer↔dimer shifts. To test the possibility of potential *redox* modulation of TOPs, we investigated the effects of dithiothreitol (DTT) which is a strong thiol-based reductant capable of modulating the activity of many *redox*-sensitive proteins (Cleland, 1964). Purified TOP1- and TOP2-His were incubated with 500 μM dithiothreitol (DTT) and passed through the Superdex 200 gel filtration column. TOPs elution profiles showed a dramatic shift toward the monomeric fractions—the approximate dimer:monomer ratio shifted to 1:15, with the second peak corresponding to the dimer being eliminated almost completely after incubation with the thiol-based reductant (Figure 2C). By comparing the total absorbance (A280) intensities of the monomer and dimer peaks before and after DTT treatment, we found that the total amount of TOP1 or TOP2 did not change after incubation with DTT, suggesting that the reduced amount of the dimer was a result of the reduction of *redox*-sensitive disulfide bonding. We conclude that thiol-based reducing conditions have the ability to directly modulate TOP dimerization, reductive conditions facilitating an increase in the monomer/dimer ratio.

Altogether, our results suggest that variations in *redox* conditions alter TOPs monomer/dimer ratio through, possibly, disruption of disulfide bonds.

### Dithiothreitol inhibits the enzymatic activity of TOP1 and TOP2

Our results so far indicate that TOP1 and TOP2 monomers and dimers co-exist in an approximately 3:1 equilibrium under physiological conditions and that the monomer/dimer ratio is modulated by the thiol-based reductant DTT. We were interested to investigate the peptidase activity of TOP1 and TOP2 monomeric and dimeric fractions and the potential effect of thiols on their activity.

**Figure 3:**
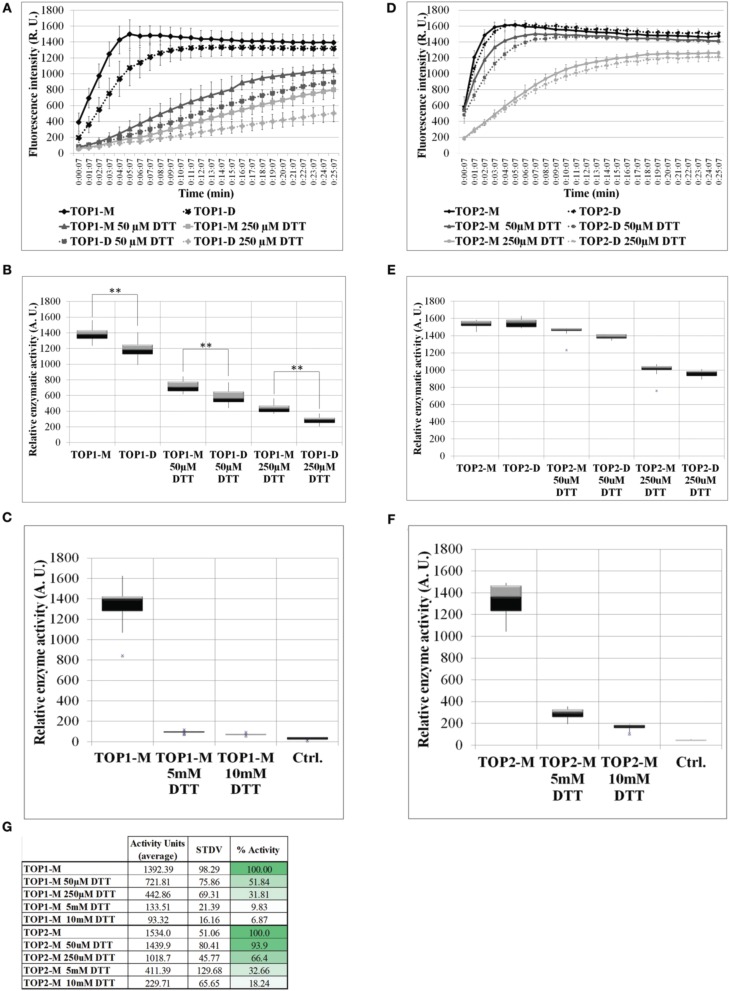
**The activity of TOP1 and TOP2 monomer or dimer in the presence or absence of dithiothreitol**. 0.1 μg of purified recombinant TOP1 or TOP2 was incubated in the reaction buffer solution containing 20 μM of the MCA-peptide. MCA-peptide emit detectable fluorescence upon cleavage. The activity was assessed at λ excitation of 328 nm and λnewline emission of 393 nm over the course of 25 min. Quantification of fluorescence intensity was expressed in the levels of fluorescence emission per minute and shown as relative enzymatic activity. **(A)** Fluorescence intensity of TOP1 monomer and dimer activity over time in reaction buffer containing no DTT or 50/250 μM DTT. **(B)** The relative enzymatic activity of TOP1 monomer and dimer after incubation in reaction buffer or reaction buffer containing 50/250 μM DTT. **(C)** The enzymatic activity of TOP1 under 5 and 10 mM DTT. Control constitutes reaction buffer containing no TOP enzyme. **(D)** The fluorescence emitted by the cleavage by TOP2 of the substrate over time. **(E)** The quantification of TOP2 monomer and dimer activity in terms of relative enzymatic activity in the presence or absence of 50/250 μM DTT. **(F)** The relative activity of TOP2 upon incubation with 5 and 10 mM DTT. Reaction buffer without TOP2 enzyme is utilized as the control. **(G)** The average relative activity and percent activity of TOP1 and TOP2 monomer under increasing concentrations of DTT.

The activity of freshly eluted TOP1-His and TOP2-His monomers and dimers was tested on a fluorogenic peptide substrate (Moreau et al., 2013) in the absence (control) or in the presence of increasing concentrations of DTT (reductive environment). Testing both the monomeric and dimeric fraction allowed us to assess the monomer↔dimer dynamics under a range of reductive conditions. TOP1 dimeric and monomeric fractions under control conditions reached the same level of activity after 10 min; however, their specific activities differed significantly over the 25 min recorded, with the dimers showing lower activity values than the monomers. Addition of 50 or 250 μM DTT inhibited the activity of TOP1 fractions up to 50% and notably, TOP1 dimers retain the lower average activity than the monomers at both DTT concentrations (Figures 3A,B,G). It may be that the inhibitory effect of the initially dimer TOP1 fraction in tandem with the inhibitory effects of DTT results in less substrate cleaved over the incubation time.

TOP2 monomers and dimers exhibited similar levels of activity under both control and reductive conditions, suggesting that unlike TOP1, TOP2 more readily achieves the monomer:dimer 3:1 equilibrium in non-reducing or reducing conditions and that the retained difference is a product of the initial inhibitory effect of the dimer on the enzyme activity in tandem with the inhibition by DTT. As with TOP1, both TOP2 monomeric and dimeric fractions were inhibited by the thiol-based reductant; TOP2 fractions maintained a higher level of activity at both 50 and 250 μM DTT—93 and 66%, respectively (Figures 3D,E,G). Further, to test TOP1 and TOP2 activity in a highly reductive environment, monomeric preparations were tested with 5 and 10 mM DTT. TOP1 maintained 10 and 7% activity, respectively, and TOP2 retained 33 and 18% activity respectively, compared to controls (Figures 3C,F,G). The difference in activity between the DTT treated monomer and untreated monomer indicates that potential intramolecular bonds may be affected by the reducing agent.

We conclude that in a thiol-driven reductive environment the activities of both TOPs are inhibited, both of the dimeric and monomeric fractions. Our data suggests that potential thiol-sensitive intra- and inter-molecular disulfide bonds in TOPs are critical for both the activity and the monomer-dimer oscillations of the proteins.

### TOP1 and TOP2 maintain their enzymatic activity in a wide range of pH

The transit peptide of TOP1 facilitates the dual transport of the protein to the chloroplast and mitochondria (Kmiec et al., 2013; Moreau et al., 2013). The function and activity of organellar enzymes is strongly influenced by pH changes in their environment caused by fluctuations in the light quality and quantity (Buchanan, 1980; Scheibe, 1991). The stroma and mitochondrial matrix typically represent an alkaline environment. The pH of the stroma fluctuates from 6.2 to 4.6 in light versus darkness (Smith and Raven, 1979). In contrast, the pH of the cytosol is more stable and centers around 7.1 (Gout et al., 1992).

**Figure 4:**
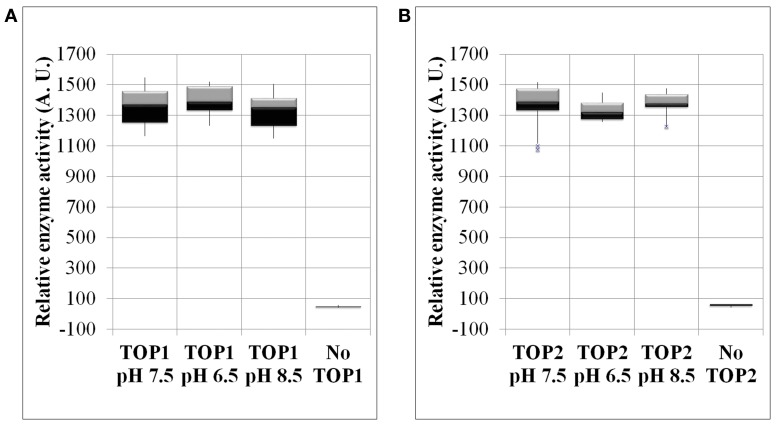
**The enzymatic activity of TOP1 and TOP2 under changing pH conditions**. Recombinant bacterial expressed TOP1 or TOP2 was incubated with 20 μM MCA-peptide under varying pH conditions. **(A)** TOP1 activity in pH values of either: 6.5, 7.5, or 8.5. **(B)** TOP2 activity in pH values 6.5, 7.5, and 8.5. In all pH experiments, the buffer solution containing the MCA-peptide in the absence of TOP was used as a control. The activity was assessed at λ excitation of 328 nm and λ emission of 393 nm over the course of 30 min. The relative enzymatic activity was calculated by the sum of the fluorescence emission per minute. The error bars are the standard deviation of 12 replicates.

To determine whether pH changes represent a potential regulatory mechanism of TOP activity, we examined the activity of recombinant TOP1- and TOP2-His under a range of pH conditions. We found that TOPs activity on the fluorogenic substrate is impervious to changes of pH both toward more acidic or basic values. TOP1 or TOP2 activity at pH 7.5 does not significantly differ from their activity at pH 6.5 or 8.5 (Figures 4A,B).

Thus, TOPs are robust enzymes with the capability of being functional in a range of pH values including values outside of the physiological pH span of the cytosol or organelles.

### *TOP1* and *TOP2* participate in the plant response to oxidative stress induced by methyl viologen

Prior evidence suggests that *TOP1* and *TOP2* peptidases contribute to plant defense against oxidative stress triggered by pathogens or abiotic factors (Polge et al., 2009; Moreau et al., 2013). We further explored the functions of *TOPs* to gain insight into the potential role of *TOP1/TOP2*-mediated pathways in ROS-triggered PCD.

Various treatments or abiotic stress conditions induce higher rates of ROS synthesis and drive their accumulation. We tested the phenotypes of *top* mutant seedlings to a panel of ROS-inducing factors including methyl viologen (MV), selenite, cadmium, antimycin A, exogenous hydrogen peroxide treatment, and salinity stress. With the exception of MV treatments, the other treatments produced no distinguishable phenotypes in *top* mutants.

MV impairs photosynthesis by interfering with electron transport of the photosystems and by generating toxic superoxide anions (Farrington et al., 1973; Härtel et al., 1992; Krieger-Liszkay et al., 2011), induces lipid peroxidation and interferes with electron transport in mitochondria (Dodge, 1971; Palmeira et al., 1995). The generation of ROS further damages the photosystems which inhibits growth, chloroplast homeostasis, and leads to PCD (Farrington et al., 1973).

**Figure 5:**
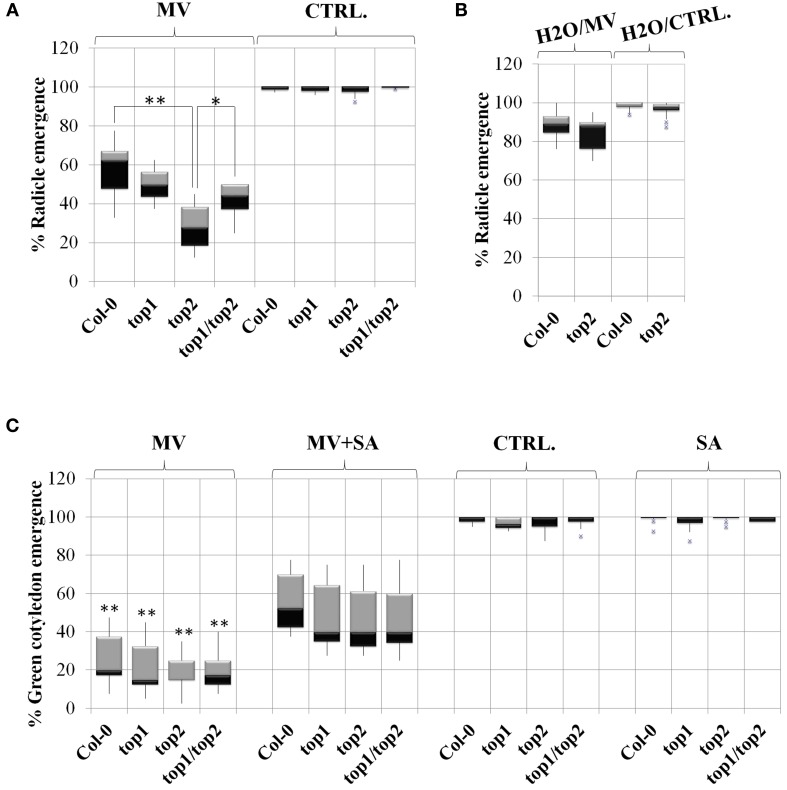
**TOPs involvement in methyl viologen stress is SA-independent**. Radicle and cotyledon emergence in the presence or absence of salicylic acid (SA), Methyl Viologen (MV) or SA and MV, in the *top* mutants described in Moreau et al. (2013). Box plots represent percentage of radicle or cotyledon emergence under various treatments as noted above each plot. **(A)** MV inhibits radicle emergence in the *top2* mutant. Seeds were stratified directly on MV-containing medium or no-MV (CTRL) media; *p*-values (p) (Student's *T*-test) were calculated as shown in the figure. **(B)** Stratification in water rescues the MV toxicity of *top2*. The seeds were stratified in water and then plated on MV (H_2_O/MV) or no-MV plates (H_2_O/ CTRL) plates. **(C)** Exogenous SA alleviates the defects in green cotyledon emergence of both the *top* mutants and wild type Col-0. In **(A,B)**, asterisks represent statistical significance (*T*-test) relative to wild type Col-0 calculated from over 6 replicates per treatment. In **(C)**, asterisks represent a statistical significance (*T*-test) of the difference between line performance in MV+SA and in MV (^*^p < 0.05 and ^**^p < 0.01).

To test the effect of MV on *top* mutants, seeds were sown on medium containing MV, stratified for 2 days at 4°C in darkness and grown in long-day (16 h) light conditions; the radicle and cotyledon emergence was assessed in both mutants and Col-0 control. First, we optimized our assay by testing multiple concentrations of MV (0.65, 7.5, 0.9, 0.95, 1, 1.5, and 2 μM). While radicle emergence could be seen at MV concentrations above 0.9 μM, green cotyledon emergence was strongly impeded at concentrations greater than 1 μM. The 0.9 μM MV condition became our established standard, as the differential effects of MV on radicle emergence was most apparent. In our assays, the percentages of radicle emergence after 2 days of growth on 0.9 μM MV-containing medium were assessed in mutants and Col-0 (Figure 5A). In the presence of MV, the emergence of radicles in *top2* mutant, but not *top1* or *top1 top2* mutants, was significantly inhibited compared to Col-0. The germination rates of *top* mutants on control medium (no-MV condition) showed no significant differences from that of Col-0 seedlings. MV applications on fully mature rosettes produced no significant differences between *top* mutants and Col-0. We conclude that *TOP2* positively modulates tolerance to MV exposure during seed germination; *TOP1* may potentially act in an opposite manner since combining *top1* and *top2* mutations partially rescued *top2* radicle hypersensitivity to MV.

MV induces ROS production during seed dormancy—short term MV treatment on dormant seeds within 6 h resulted in improved germination rates by breaking dormancy (Farrington et al., 1973). To determine whether the oxidative stress resulting from ROS production upon prolonged exposure (48 h) to MV during seed dormancy may be the cause of the hypersusceptibile phenotype of the *top* mutants, *top2* and Col-0 were no longer stratified in the presence of MV. Instead, Col-0 and *top2* were stratified on water for 2 days and then seeded on MV-containing plates. We found that under these conditions the germination rate of *top2* increased drastically so that the difference between *top2* and Col-0 germination was no longer significant (Figure 5B). Thus, the MV-mediated inhibition of the germination rate of *top2* only occurs when *top2* is exposed to the oxidative stress inducer during seed dormancy.

Altogether, our data indicates that *TOP1* and *TOP2* specifically mediate plant responses to MV during early development via, at least partially, distinct pathways; also, it suggest that *TOPs* do not have broad, unspecific, roles in mediating plant's oxidative stress response.

### Exogenous SA alleviates the MV toxicity in a *TOP*-independent manner

Low levels of exogenous SA lessened the damage caused by oxidative stress through the modulation of antioxidant-related activities (Lee et al., 2010) and alleviated the effects of MV on photosynthesis (Ananieva et al., 2002). In addition, our previous work indicated a connection between *TOP1, TOP2*, and SA-mediated signaling (Moreau et al., 2013). Thus, the possibility emerged that *TOPs* may contribute to the plant response to MV through an SA-mediated pathway.

To test this hypothesis, we sowed mutants and Col-0 seedlings on plates in the presence or absence of 10 μM SA and/or 0.95 μM MV and quantified their effects on the germination rate (Figure 5C). The quantification of cotyledon emergence instead of radicle emergence was done to assess the recovery effect of SA on photosynthesis. Seedlings were grown only in the presence of SA to determine its independent effect on germination. We found that all lines exhibited normal germination rates in the presence of 10 μM SA, as determined by measuring the emergence of green cotyledons indicating that, at this low concentration, exogenous SA does not impede germination. Next, we examined whether SA-induced signaling is functional in a *top* background by measuring the percentages of green cotyledons of seedlings grown in the presence of both 0.95 μM MV and 10 μM SA. We found that SA alleviated the negative effect of MV on photosynthesis on all lines to a similar extent; in average, the green cotyledon emergence rate of all lines increased by 45%. Thus, the SA-mediated signaling triggered by low amounts of exogenous SA is unaffected in *top* mutant background.

Taken together, our results suggest that the SA-mediated pathways activated by MV exposure function independently or are genetically downstream of the *TOP* pathways.

### A model of TOP-mediated cellular functions in oxidative stress

**Figure 6:**
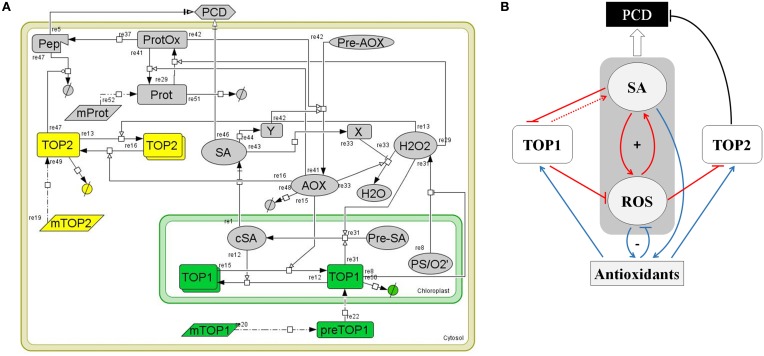
**A computational model of *TOP1* and *TOP2* functions in the oxidative stress response. (A)** Graphical representation of the TOP model. The model represents a plant cell containing various cellular elements (transcripts, proteins, small molecules, peptides) linked by biochemical reactions (transcription, translation, transport, association/disassociation, inhibition, and catalysis) and the kinetic laws associated with the biochemical reactions. The model was build using *CellDesigner 4.4* software. **(B)** Logical diagram visualizing the relationships among TOP1, TOP2, SA, ROS, and Antioxidants in the context of the oxidative stress response. Pathways active during the oxidative burst phase are in red, pathways active during the reductive burst phase are in blue, and PCD-triggering pathways are in black. The symbol “+” indicates the SA-ROS positive feed-back loop; “−” shows the negative feed-back between ROS-Antioxidants. The punctuated line arrow depicts the hypothesized function of TOP1 in SA synthesis.

To understand how *TOP1*- and *TO2*-mediated pathways operate and influence each other in the context of SA signaling and stress response, we undertook an analytical approach to study their system-level dynamics. We developed a systems biology model that characterizes TOP1 and TOP2 functions in the context of the SA- and *redox*-triggered PCD (Figure 6A). The model was built by integrating experimental observations from the analysis of *top* mutants, biochemical analysis of TOPs and current knowledge on *SA* and the oxidative stress response pathways; the measurable cellular phenotype integrated in the model is the PCD. The architecture of the model relies on the relation between five main components: SA, ROS (H_2_O_2_), antioxidants (AOX), and TOP1/TOP2. Following is a description of the model development that includes the rationale for selecting molecular species, reactions, rate equations, and parameters.

#### SA in the context of redox homeostasis and PCD signaling

The core of the model is constituted by SA, ROS (H_2_O_2_), and AOX and their relationships. We included in the model the functions of SA in rapport to TOPs from Moreau et al. (2013) and the present study, pathways that represent the biochemical reactions related to production, degradation, and signaling functions of H_2_O_2_, and the cellular activities of antioxidants as symbolized by a generic AOX molecule. A large number of reactions related to antioxidant activities are known, however we omitted biochemical activities of specific AOX on TOPs as they are not yet understood. The main pathways that drive the SA, ROS, and AOX activities are described by the following reactions:

- The central regulators of the cellular redox homeostasis are antioxidant enzymes and small MW species (such as glutathione, ascorbate, and tocopherol), which participate in cellular detoxification through scavenging of ROS, reducing oxidized thiols, and functioning as *redox* buffers (*re33* and *re41*). Catalases constitute an important part of the plant's antioxidant system; SA inhibits the activity of catalases (Conrath et al., 1995; Rüffer et al., 1995) (*re43, re33*). ROS signaling mediates the activation of the antioxidant system (*re29, re42*).
- SA synthesized in the chloroplasts (cSA) is transported into the cytosol (SA) (*re1*) (Fragniere et al., 2011; Serrano et al., 2013); H_2_O_2_ the most abundant ROS species produced from superoxide during photosynthesis diffuses and/or is transported across chloroplastic membranes (*re8*) (Bienert et al., 2006, 2007; Mubarakshina et al., 2010). The reduced form of glutathione (GSH) maintains a reductive environment in the cell (Han et al., 2013). GSH-dependent Glutathione Peroxidase catalyzes hydrogen peroxide detoxification and forms GSSG, the oxidized form of glutathione. Glutathione reductase (*GR*) catalyzes the reduction of GSSG to GSH and helps maintain a reducing cellular milieu (Meyer et al., 2012; Deponte, 2013). Accumulation of H_2_O_2_ regulates increases GSH/GSSG which in turn activates the Isochorismate syntase1 (ICS1)-dependent SA synthesis (Han et al., 2013) (*re31*).
- *Redox* signals that drive the development of PCD (*re29, re37*, and *re5*) may be transduced via thiol-driven post-translational modifications in sensor proteins with higher chemical reactivity (Mou et al., 2003; Apel and Hirt, 2004; Buchanan and Balmer, 2005; Temple et al., 2005; D'Autreaux and Toledano, 2007). Such sensors contain residues whose location and ionic state render them sensitive to oxidation (Ghezzi et al., 2005; Nagy, 2013). *Re29* models the positive effect of ROS accumulation on the reversible oxidation of protein sensors (*ProtOx*), while antioxidant enzymes, such as the GSH-dependent glutaredoxins, catalyze the reverse reaction (Rouhier, 2010) (*re41*). Under high oxidative stress or in mutants with a defective proteasome pathway, the accumulation of a partially degraded peptide (*re37*) triggers PCD through (*re5*). An independent SA-driven PCD pathway (*re46*), involves a transcriptional response via *NPRs* (Hoeberichts and Woltering, 2003; Jayakannan et al., 2015).
- Antioxidant production is enhanced by SA (*re42*) though a transcriptional pathway (*re44*). This simulates the SA modulatory effect on the GSH/GSSG ratio—SA increases GSH cellular content by enhancing the transcription of enzymes in the glutathione cycle (Li et al., 2013).

#### *TOP1/TOP2* pathways

- *TOPs* are nuclear-encoded genes; in the model, the respective mRNAs (*mTOP1* and *mTOP2*) are translated into proteins *(re20* and *re19*).
- *TOP* proteins have distinct subcellular localizations; *re20, re22*, and *re50* summarize the maturation, import from cytosol into the chloroplast and degradation of *TOP1*, while *re19* and *re49* describes the synthesis and degradation of cytosol-localized *TOP2* (Kmiec et al., 2013; Moreau et al., 2013).
- We hypothesized that SA and AOX modulate the enzymatic activities of TOP1 and TOP2 by adjusting the monomer/dimer ratios of TOP1 and TOP2. Specifically, the chloroplastic SA binds TOP1 and inhibits its activity by decreasing the monomer/dimer ratio (Figure 1; Moreau et al., 2013) (*re12*) while the AOXs' reducing activity increases TOP1's monomer/dimer ratio (*re15*) by promoting monomerization. Likewise, TOP2 activity is modulated by shifts in the AOX *redox* status whereby a reductive environment favors monomerization (*re16*) and increases TOP2 activity, while an oxidative environment favors TOP2 dimerization (*re13*) and inhibits its proteolytic activity (*re47*) (Figures 2, 3).
- TOP1 and TOP2 sustain organelle- and cytosol-specific proteolytic pathways respectively. We postulate that TOP1 activity sustains normal levels of ROS accumulation in chloroplasts (*re8*), possibly by participating in chloroplasts import of antioxidant enzymes (e.g., GPX) or degradation of oxidized proteins (Kmiec et al., 2013). We postulate that TOP1 activity faciliates SA accumulation by participating in the import of enzymes that catalyze SA synthesis (*re31*). On the other hand, TOP2 may modulate the execution of PCD as part of a cytosolic proteolytic pathway activated by MV or other factors causing oxidative stress (Polge et al., 2009). We hypothesize that TOP2 controls the accumulation of a signaling peptide—a positive regulator of PCD (*re5*); thus, the irreversible oxidation of proteins leads to their degradation via the proteosomal machinery (*re37*) and TOP2 (*re47*).

#### Species, reactions, and selection of parameters

The model contains 22 molecular species (3 mRNAs, 8 small molecules, and 11 proteins), 25 reactions and 44 reaction parameters, described in Supplemental Tables S1–S3. We selected the species' initial values based on the published literature: SA (cSA) basal levels were selected in the interval (0.05–1 μM) (Enyedi et al., 1992; Abreu and Munné-Bosch, 2009); H_2_O_2_ levels within (1–100 μM) (Veljovic-Jovanovic et al., 2002) about 40-Jovanmol g^−g^ FW; and AOX levels within (1–100 μM). TOP1 and TOP2 expression was normalized to maintain a ratio of 1/2, with TOP1 expressed at 1/10 level in comparison to the large PS-I complex as a baseline, based on Genevestigator data (Hruz et al., 2008). The initial monomer:dimer ratio of TOPs was chosen to be 3:1 as observed in our assays. Molecules that are consumed during the simulation of the cellular stress phenotype (*mTOP1, mTOP2, mProt, Pre-SA, Pre-AOX, Prot*) were selected to the normalized value of 1 to limit their impact on the dynamics of TOPs pathways. The concentration of superoxide (*O*^•−^_2_) was the input variable for controlling the oxidative stress. The remaining species (*Pep, X, Y*) were initialized to zero or low concentrations.

Several reactions (*re12, re13, re15, re16, re19, re20, re29, re41, re47, re48, re31, re42*, and partially *re33*) follow a Michelis-Menten rate law; the inhibitor has the rate equation: k^*^_1_E^*^S/(*k*_2_+*S*) (*E*-enzyme, *S*-substrate); the PCD-trigger reactions (*re5* and *re46*) were modeled with a Hill dynamics (k^*^_1_*S^n^*/(*k^n^*_2_+*S^n^*)) and the Hill coefficient (*n* = *2*). Simple reactions (protein production, degradation, and transport) follow a mass action rate law (*re1, re22, re37, re43, re44, re49, re50, re51, re52*). The rate law for TOP1 inhibition of H_2_O_2_ production (*re8*) and catalysis of H_2_O_2_ reduction to H_2_O (*re33*) is: k_1_*S/(1+E/k_2_). The production of AOX (*re42*) was modeled by the addition of two independent reactions, one driven by SA and the other by the level of cellular oxidative stress (*ProtOx*). The reduction of H_2_O_2_ to H_2_O (*re33*) was also obtained through two independent pathways—one driven by AOX and another inhibited by SA through a transcriptional pathway involving an unknown protein species (X) (Rao et al., 1997). The synthesis of cSA from precursors was modeled as a single pathway regulated by both H_2_O_2_ through the GSH/GSSG system and TOP1, probably through its proteolytic activity necessary to import and mature enzymes involved in SA synthesis. A background rate of SA synthesis independent of TOP1 activity was also necessary to explain the experimentally observed mild MV phenotype in *top1*.

Reactions parameters were selected such that enzymatic reactions occur at high rates (normalized to *k* = 1.0 s^−1^ or *k_cat_* = 1.0 s^−1^) while reactions involving transcriptional control have one order of magnitude slower rates; the Michaelis constant was selected by default (*k_M_* = 1 μM) and was varied between 1 and 10 μM subsequently. The rate constants for the mass action kinetics describing protein production/degradation were chosen two orders of magnitude lower than the enzymatic reactions (*k* = 0.01 s^−1^). Since quantitative time series data for the reactions were not available, the parameters were initially selected as described above. Subsequently, a parameter scan was performed to observe the dynamics of species and reaction fluxes; the parameters were adjusted to fit the observed dynamics of the stress phenotypes of *top* mutants, of the ROS-AOX inhibition and SA-ROS amplification loops, and the hallmark aspects of the oxidative stress response—an initial oxidative wave followed by a strong reductive phase. We adjusted the reaction rates to establish the observed monomer/dimer ratio and to equilibrate the production and degradation of species. Supplemental Table S3 lists the parameters for which PCD, ROS, AOX, and SA dynamics is robust and correlates with *top* phenotypes.

### TOP model dynamics and analysis of oxidative stress phenotypes

The computational model in Figure 6A was summarized in a logical diagram (Figure 6B). The diagram contains the SA-ROS amplification loop driving the oxidation wave, the AOX wave stimulated by the SA accumulation, and the ROS-AOX inhibitory loop driving the *redox* reductive wave and the connected TOP1/TOP2 pathways. The expected activity and dynamics associated with the TOPs is as follows. We hypothesize that in the oxidative burst phase TOP1 has a positive control over the SA-ROS amplification loop, driving a higher SA accumulation while limiting the ROS accumulation in the chloroplast; in the reductive phase, TOP1 activity is enhanced via monomerization under the enzymatic activity of antioxidants—SA drives antioxidants that increase TOP1 activity which reduce ROS accumulation. On the other hand, in the oxidative burst phase the amount of TOP2 monomers decreases and, in consequence, TOP2 activity is inhibited which may trigger PCD via accumulation of oxidized products; the amplitude of the PCD correlates with the amplitude of the oxidative burst; in the reductive phase TOP2 increases in activity (via monomerization) suppressing the SA-independent pathway leading to PCD.

**Figure 7:**
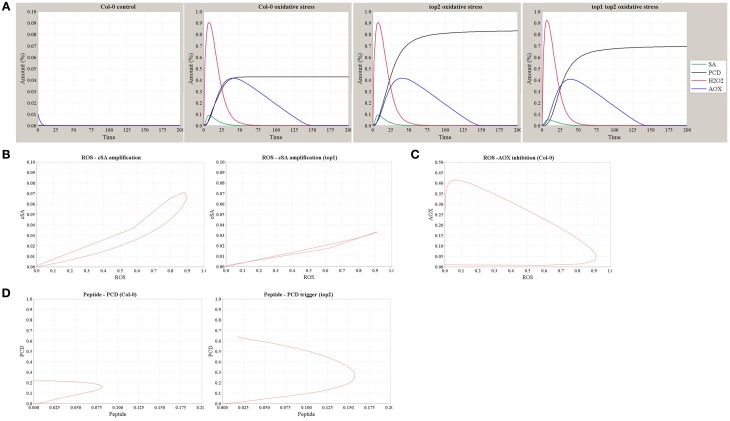
**Simulation outputs of the computational model of *TOP1* and *TOP2* functions in the oxidative stress response. (A)** The dynamics of SA, H_2_O_2_, AOX, and PCD simulated in the Col-0 control (no MV stress) and in Col-0, *top2*, and *top1 top2* mutants in conditions of MV-induced oxidative stress. **(B)** The dynamics of ROS-SA amplification loop in Col-0 and the impaired amplification in *top1* mutant. **(C)** The dynamics of ROS-AOX inhibition loop under oxidative stress in Col-0. **(D)** The dynamics of PCD triggering and peptide accumulation in Col-0 and *top2* mutant.

Using the model shown in Figure 6A, we simulated the dynamics of SA, H_2_O_2_, AOX, and the resultant PCD in Col-0, *top2*, and *top1 top2* backgrounds in conditions of MV-induced oxidative stress or the stress-free control (Figure 7A). Increased superoxide levels resulted in a rapid oxidative wave, which increased SA amount via the SA-ROS amplification loop and subsequently triggered an increase in the AOX accumulation and a longer reductive wave. The intensity of PCD caused by oxidative stress is determined by the level of SA accumulation—which is lower in *top1* (Figure 7B), and the accumulation of un-degraded peptide *Pep*—which reaches a higher level in *top2*. Figure 7C details the dynamics of ROS-AOX inhibition loop under oxidative stress (MV challenge): the initial fast increase in ROS accumulation triggers a gradual increase in the AOX level which negatively regulates the ROS and suppresses the oxidative burst. Furthermore, simulation outputs reproduce the observed *top2* MV hypersensitivity; accumulation of partially degraded peptides in *top2* results in a stronger PCD phenotype (Figure 7D). Thus, the model explains the milder *top1 top2* MV phenotype compared to *top2* by predicting lower levels of *SA-ROS* amplification in *top1 top2* where the TOP1-amplified oxidative burst does not function at full capacity and thus, partially alleviates the higher PCD levels of *top2*.

Taken together, we demonstrated that the expected dynamics and stress phenotypes of *top* mutants were correctly predicted by this qualitative model of TOP1 and TOP2 pathways.

### Discussion

In this study, we investigate the characteristics, enzymatic properties, and cellular roles of *TOP1* and *TOP2* as they relate to the plant oxidative stress response. We generated a theoretical model of the functions of *TOP1* and *TOP2* in the context of an essential set of cellular elements and processes, deduced by integrating experimental data from the analysis of *top* mutants and the biochemical characterization of TOP proteins.

We determined that TOP1 and TOP2 are capable of self- and hetero-dimerization using two distinct systems: the *in vivo* protoplast system, which accounts for factors that may modulate TOPs interactions such as the cellular *redox* environment and potential post-translational modifications, and *in vitro* gel chromatography, a method using purified proteins which allows for fine adjustments of the assay conditions (Figures 1, 2). Interestingly, TOP2 dimers showed a higher interactions affinity than TOP1 dimers in this assays. It is notable that TOP2 has one additional Cys residue in the peptidase domain compared to the chloroplastic TOP1; it is possible, that similar to the case of the poplar thioredoxins (Chibani et al., 2012), this Cys residue may be essential for TOP2's ability to form tighter dimers, and may represent an evolutionary advantage in the highly reducing conditions of the cytosol.

TOP2 dimerization was influenced by treatments with SA and the thiol-based reductant DTT. We demonstrated that incubation of protoplasts with exogenous SA destabilized the TOP2 dimer. This result could be the effects of direct binding of TOP2 to SA, however this hypothesis is unlikely as we previously showed that TOP2-SA association has a low affinity and thus, is probably functionally irrelevant (Moreau et al., 2013). Alternatively, SA's effects on TOP2 self-association may be explained by the indirect consequences of SA treatment on the *redox* homeostasis of the protoplasts, akin to NPR1 monomerization and thioredoxins activation (Mou et al., 2003; Tada et al., 2008). Several observations strengthen the latter hypothesis: (1) we observed a concentration-dependent effect of SA on TOP2 dimerization suggesting that the inhibition of TOP2 dimerization may occur via disruption of multiple intra- or intermolecular disulfide bonds by the SA-mediated increase in the reductive potential of the protoplasts; (2) DTT treatment of purified TOP2 shifted the monomer/dimer ratio from 3:1 to 15:1 (Figure 2), indicating that a reductive environment destabilizes disulfide bridges and favors accumulation of TOP monomers; and (3) The effects of SA on TOPs dimerization is in contrast to its effect on the MKK5-HOPF2, an interaction not known to be mediated by disulfide bridges and which was drastically reduced by the lowest SA amount tested.

Given our results, it is plausible that *redox* changes induced by SA can potentially influence the TOPs monomer-dimer equilibrium. We postulate that SA-mediated *redox* shifts may lead either to dimerization during the oxidizing burst or monomerization during the reductive bursts. It is possible that direct or indirect effects of SA on TOPs' monomerization/dimerization state are further regulated by additional factors such as the SA local concentrations, TOPs sub-cellular localization and the temporal and compartment-specific redox environments following stress driven by the major glutathione-ascorbate antioxidant systems—all aspects that require further investigation. Altogether, our results support the hypothesis that TOPs are regulated on the basis of cellular *redox* state.

We examined the activity of TOP1 and TOP2 monomers and dimers to understand if TOPs self-associations and thiol-sensitivity have regulatory roles. Notably, TOP1 and TOP2 show a similar trend of sensitivity to the metazoan TOP toward high concentrations of DTT (5 and 10 mM DTT) indicating that akin to the proposed effects for the metazoan TOP (Lew et al., 1995; Shrimpton et al., 1997), high levels of DTT inhibit TOP1 and TOP2 by interfering with the zinc co-factor binding and/or disrupting intramolecular bonds. Remarkably, plant TOPs differ from the metazoan counterpart in one important aspect; unlike metazoan TOP, TOP1, and TOP2 activity is inhibited by low DTT concentrations (below 1 mM). While metazoan TOPs are Cys-rich proteins, the comparatively lower number of Cys in TOP1 and TOP2 may potentially result in fewer intra- and intermolecular disulfide bonds, or, alternatively, form bonds that may be more accessible to reduction; in either case, the reduction of the intramolecular bonds may be deleterious to the structure of the monomeric TOPs and DTT able to limit their activity even at low concentrations. Altogether, our results on the activity levels displayed by various TOP1/TOP2 fractions in the absence or presence of DTT, alongside published studies on metazoan TOPs, lead us to hypothesize that TOPs contain disulfide bonds and that monomers are the active forms, while the dimers and potential multimers are inactive enzymatically.

*TOPs* functions in modulating pathogen-induced PCD (Moreau et al., 2013) and the newly-uncovered biochemical characteristics of TOPs motivated us to investigate their particular contributions to the plant oxidative stress response. The oxidative triggers modifications in the proteome composition and activity and *TOPs* unique attributes and functions render them likely participants in the *redox*-mediated signaling. A prior study on plant *TOPs* suggested their role in limiting oxidative damage following heavy metal stress (Polge et al., 2009). Although we have not detected any unusual responses of *top* mutants to heavy metal stress, we provide evidence that functional *TOPs* are required to mediate the damage caused by MV, a potent inducer of photo-oxidative stress. MV readily interferes with the photosystems resulting in severe chloroplastic-derived oxidative stress (Babbs et al., 1989). ROS accumulation is known to promote seed germination (Marino et al., 2012) and specifically, short term exposure to MV increased ROS levels and interrupted dormancy in *Arabidopsis* and *Helianthus* seeds (Oracz et al., 2007; Leymarie et al., 2012). Interestingly, *top2* responded with increased sensitivity to MV when scoring for radicle emergence indicating that the cytosolic TOP2, rather than the chloroplastic TOP1, is the critical *TOP* for MV tolerance in this organ. The importance of the cytosolic TOP2, alongside the chloroplastic TOP1, for the plants' MV tolerance is in line with studies indicating that chloroplastic stress induces expression of both cytosolic and chloroplastic antioxidant enzymes and supporting the role of cytosol as a major site for detoxification systems associated with photosynthesis (Mullineaux et al., 2000; Yabuta et al., 2004).

SA alleviated the germination of *top* mutants and Col-0 seeds grown in the presence of MV. The protective properties of treatments with physiological concentrations of SA are considered a general consequence of SA's antioxidant activities as evidenced by lowered cellular ROS or nitric oxide levels in *Arabidopsis* and crops, monocots and dicots, subjected to abiotic or biotic stress upon exogenous SA application (Lee et al., 2010; Gémes et al., 2011; Zhang et al., 2011; Wang and Liu, 2012). It is possible that a similar mechanism is responsible for relieving MV toxicity in *top* seeds. While the precise function of *TOPs* in modulating MV tolerance is unclear, it is likely that *TOPs* have ROS-protective roles by cleaving oxidized peptides and preventing their accumulation in the cytosol or chloroplast; in parallel, TOPs may indirectly participate in the ROS-mediated signaling by cleaving/degrading specific peptides with signaling properties that modulate PCD.

A qualitative model was constructed describing TOP1 and TOP2 functions in the development of PCD associated with oxidative stress, in the context of known SA- and *redox*-mediated signaling pathways (Figures 6, 7). TOP1/TOP2 functions are described within the SA-ROS-Antioxidant framework. Perception of both abiotic and biotic stress triggers shifts in the *redox* potential of the cellular milieu, primarily driven by increases in the endogenous concentration of SA and ROS, which are critical for the downstream signal transduction and induction of defense transcriptome and metabolome. Two distinct *redox* phases have been described: (1) the oxidative burst, driven by fast reactions initiated by ROS, is controlled by the interplay between SA and ROS synthesis (Leon et al., 1995; Rao et al., 1997; Shirasu et al., 1997); (2) the reductive burst, which is a consequence of the oxidative burst and is regulated by SA and synthesis of antioxidants (Mou et al., 2003; Tada et al., 2008). We postulate that TOP1 activity of attenuating ROS increase is repressed by high SA accumulation during the oxidative phase and enhanced by the antioxidant actions during the reductive phase; on the other hand, TOP2—less active during the oxidative phase and more active during the reductive phase—is regulated by, but not a contributor to, the SA-ROS-Antioxidant driven *redox* oscillations.

Altogether, our study that combines analytical and experimental approaches supports the hypothesis that the interplay between TOP1-controlled chloroplastic events and the cytosolic TOP2 modulates the development of PCD through the ROS-SA-AOX axis.

Further studies of TOP1 and TOP2 effects on the plant oxidome alongside identification of their respective peptide substrates are required to validate or reject the hypotheses presented in the qualitative model. Undeniably, the SA signaling network during the biotic/abiotic stress comprises numerous elements with complex interactions; it would be a challenging task to assemble a quantitative model comprising all components and biochemical reactions and to demonstrate their precise spatial and temporal control.

## Materials and methods

### Germination treatments

The germination experiments were done on petri plates containing 35 mL of MS agar containing MV and/or SA. Germination on MS agar plates containing no MV or SA was used as controls. Seeds were either stratified directly on plates unless indicated otherwise. After stratification of 2 days in darkness, the seed were germinated under 16 h light/8 h dark conditions. Germination was counted by either assessing radicle emergence after 2 days or green cotyledon emergence after 5 days.

### Split-luciferase complementation assays

Coding sequences of *TOP1* and *TOP2* were cloned into the pENTR vector. Fusion to N-terminal or C-terminal luciferase protein was done by their subsequent sub-cloning into pDuEx-DC6 and pDuEx-AC6 (Fujikawa and Kato, 2007). Vectors were transformed into DH5 alpha *E. coli*. *MKK5* and *HOPF2* were cloned similarly and utilized as a positive control. Plasmid was extracted using Zymo's Zyppy Maxi Plasmid Prep kits (Zymo Research). Protoplast co-transformation with plasmids was done in 96 well plates using a modified protoplast extraction and PEG transformation protocol derived from Yoo et al. (2007), Wu et al. (2009) and Singh et al. (2014). At 12–16 h post-transformation, the protoplasts were incubated with the luciferase substrate in the presence or absence of SA. Luminescence was detected in a Synergy Microwell plate reader.

### Protein purification

His-tagged TOP1 was expressed in pET-28(a) vector in BL21. His-Tagged TOP2 was expressed in pET-32 vector in BL21. After the induction of His-tagged protein production, a crude extract of proteins were subjected to HisPur Cobalt Column purification (Thermo Scientific). The proteins were further purified using size-exclusion chromatography. Additional purification and concentrations were done using the Amicon Ultra 50 k Centrifuge Filter (EMD Millipore) in the case of the monomer. Pierce Concentrators 150 k MWCO (Thermo Scientific) was used to further purify and concentration the dimer. Small aliquots were stored or utilized in the same day.

### Enzymatic activity

Measurements of TOP activity was done by utilizing Synergy 4 micro well reader with the capacity of λ excitation of 328 nm and λ emission of 393 nm. 0.1 μg purified peptidase was incubated in a 99 μL reaction solution contacting TRIS-HCl buffer and 20 μM of the fluorogenic peptide substrate. The fluorogenic substrate, Mca-Arg-Pro-Pro-Gly-Phe-Ser-Ala-Phe-Lys (Dnp)-OH (Enzo Life Sciences), emits detectable fluorescence upon cleavage. Unless stated otherwise, the pH of the reaction mix was 7.5.

### Gel filtration chromatography

Proteins eluted from cobalt column was subjected to size exclusion chromatography. Maximum volume of 4 mL of the eluted extract were injected to the gel filtration apparatus into the Superdex 200 column. The filtration apparatus facilitated the elution of cobalt purified protein based on size. UV at absorbance 280 nm while elution the respective fractions allowed the quantification of protein concentration within the sample. In the case of the DTT treated TOP samples, the cobalt TOP extractions were incubated with 500 μM DTT overnight and then subjected to size-exclusion chromatography.

### Systems biology modeling and simulation

The model was described in System Biology Mark-up language (SBML) format and analyzed using Cell Designer 4.4 (Funahashi et al., 2006) simulation tools. Model stability was assessed by observing stability over a range of parameters for species concentrations and reaction constants. The model was stable in the parameter range studied. The SBML model is available upon request.

### Conflict of interest statement

The authors declare that the research was conducted in the absence of any commercial or financial relationships that could be construed as a potential conflict of interest.

## Supplementary material

Table S1 **Model variables**.

Table S2 **Model reactions**.

Table S3 **Model parameters**.
