# Supplementary material for: Dimerization and thiol sensitivity of the salicylic acid binding thimet oligopeptidases TOP1 and TOP2 define their functions in redox-sensitive cellular pathways
Source: Front Plant Sci. 2015 May 18;6:327. doi: 10.3389/fpls.2015.00327 (PMC4434903; doi:10.3389/fpls.2015.00327)
Supplement: Table S1 — Model variables. [file Table1.PDF]

Table 1. Model Species.

Class	ID	Name	Compartment	Quantity	Initial quantity	Substance Units	Description
SIMPLE_MOLECULE	s1	SA	Cytosol	Concentration	0	µM	Salicylic acid
SIMPLE_MOLECULE	s5	cSA	Chloroplast	Concentration	0.001	µM	Chloroplastic SA
PROTEIN	s7	TOP1	Chloroplast	Concentration	0.2	µM	TOP1 monomer
PHENOTYPE	s13	PCD	default	Concentration	0	dimensionless	Programmed Cell Death
PROTEIN	s15	TOP2	Cytosol	Concentration	0.37	µM	TOP2 monomer
PROTEIN	s19	TOP1	Chloroplast	Concentration	0.09	µM	TOP1 dimer
PROTEIN	s20	TOP2	Cytosol	Concentration	0.2	µM	TOP2 dimer
RNA	s23	mTOP1	Cytosol	Concentration	0.91	µM	TOP1 mRNA
RNA	s24	mTOP2	Cytosol	Concentration	0.9	µM	TOP2 mRNA
PROTEIN	s25	preTOP1	Cytosol	Concentration	0.27	µM	Precursor TOP1
PROTEIN	s3	ProtOx	Cytosol	Concentration	0	µM	Oxidized protein
PROTEIN	s29	Prot	Cytosol	Concentration	1	µM	Reduced protein
SIMPLE_MOLECULE	s32	H2O	Cytosol	Concentration	1	µM	Water
DEGRADED	s34	s34	Cytosol	Concentration	0	µM	Degradation product of Pep
SIMPLE_MOLECULE	s43	Pre-SA	Chloroplast	Concentration	1	µM	Precursor of SA
SIMPLE_MOLECULE	s42	O2'	Chloroplast	Concentration	0.5	µM	Superoxide product from Photosynthesis
SIMPLE_MOLECULE	s2	H2O2	Cytosol	Concentration	0	µM	Hydrogen Peroxide
SIMPLE_MOLECULE	s35	AOX	Cytosol	Concentration	0.01	µM	Antioxidants
PROTEIN	s51	Pep	Cytosol	Concentration	0.001	µM	Peptide
SIMPLE_MOLECULE	s48	Pre-AOX	Cytosol	Concentration	1	µM	Precursors of Antioxidants
PROTEIN	s50	Y	Cytosol	Concentration	0	µM	Protein Y
PROTEIN	s49	X	Cytosol	Concentration	0.1	µM	Protein X

DEGRADED	s52	s52	Cytosol	Concentration	0	µM	Degradation product of AOX
DEGRADED	s53	sa14_degraded	Cytosol	Concentration	0	µM	Degradation product of TOP2
DEGRADED	s54	sa7_degraded	Chloroplast	Concentration	0	µM	Degradation product of TOP1
DEGRADED	s55	sa29_degraded	Cytosol	Concentration	0	µM	Degradation product of Pep
RNA	s56	mProt	Cytosol	Concentration	1	µM	Prot mRNA
