# Supplementary material for: Dimerization and thiol sensitivity of the salicylic acid binding thimet oligopeptidases TOP1 and TOP2 define their functions in redox-sensitive cellular pathways
Source: Front Plant Sci. 2015 May 18;6:327. doi: 10.3389/fpls.2015.00327 (PMC4434903; doi:10.3389/fpls.2015.00327)
Supplement: Table S2 — Model reactions. [file Table2.PDF]

Table 2. Model Reactions

Type of Reaction	ID	Reactant	Products	Modifiers	Math	Description
TRANSPORT	re1	s5	s1		s5 * k1	Transport of the chloroplastic SA to the cytosol
REDUCED_TRIGGER	re5	s51	s13		k1 / (pow(k2 / s51, 2) + 1)	Trigger of PCD by the signaling peptide Pep
STATE_TRANSITION	re8	s42	s2	s7	k1 * s42 / (1 + s7 / k2)	Production and transport of chloroplastic ROS to the cytosol under the negative control of TOP1
STATE_TRANSITION	re12	s7	s19	s5	k1 * s7 * s5 / (s7 + k2)	Dimerization of TOP1 under positive control of chloroplastic SA
STATE_TRANSITION	re13	s15	s20	s2	k1 * s2 * s15 / (s15 + k2)	Dimerization of TOP2 under positive control of hydrogen peroxide
STATE_TRANSITION	re15	s19	s7	s35	k1 * s19 * s35 / (s19 + k2)	Dissociation of TOP1 dimer to monomers under positive control of Antioxidants (AOX)
STATE_TRANSITION	re16	s20	s15	s35	k1 * s35 * s20 / (s20 + k2)	Dissociation of TOP2 dimer to monomers under positive control of Antioxidants (AOX)
TRANSLATION	re19	s24	s15		v1 * s24 / (k2 + s24)	Translation and maturation of TOP2 from the *mTOP2*.
TRANSLATION	re20	s23	s25		v1 * s23 / (k2 + s23)	Translation and maturation of TOP1
TRANSPORT	re22	s25	s7		s25 * k1	Import of precursor TOP1 in the chloroplast
STATE_TRANSITION	re29	s29	s3	s2	k1 * s2 * s29 / (s29 + k2)	Reversible protein oxidation under the positive control of hydrogen peroxide

STATE_TRANSITION	re31	s43	s5	s7,s2	s43 * k1 * (s2 / (s2 + k2)) * (k4 + s7 / (s7 + k3))	SA synthesis from precursors (preSA) via *ICS1* (*Isochorismate synthase1*) pathway
STATE_TRANSITION	re33	s2	s32	s35,s49	k1 * s2 * (s35 / (s35 + k3)) * (1 / (1 + s49 / k2))	Hydrogen peroxide detoxification to water under the negative regulation of SA, via a signaling pathway involving X component.
STATE_TRANSITION	re37	s3	s51		k1 * s3	Cleavage of oxidized proteins (ProteinOx) to form peptide (Pep) as part of the proteasomal pathway.
STATE_TRANSITION	re41	s3	s29	s35	k1 * s3 * s35 / (s3 + k2)	Reduction of oxidized proteins driven by antioxidants
STATE_TRANSITION	re42	s48	s35	s3,s50	k1 * s48 * (s50 / (s50 + k2)) + s3 / (s3 + k3)	Antioxidant synthesis under positive regulation of SA via signaling pathway containing Y element and a pathway dependent on *redox* protein sensors.
STATE_TRANSITION	re43	s1	s49		k1 * s1	SA drives hydrogen peroxide detoxification via X
STATE_TRANSITION	re44	s1	s50		k1 * s1	SA drives antioxidant synthesis via Y
TRIGGER	re46	s1	s13		k1 / (pow(k2 / s1, 2) + 1)	Antioxidant-dependent pathway triggers PCD
STATE_TRANSITION	re47	s51	s34	s15	k1 * s15 * s51 / (k2 + s51)	Pep degradation catalyzed by TOP2 monomer
STATE_TRANSITION	re48	s35	s52		k1 * s35 / (k2 + s35)	Antioxidant degradation
STATE_TRANSITION	re49	s15	s53		s15 * k1	TOP2 degradation
STATE_TRANSITION	re50	s7	s54		s7 * k1	TOP1 degradation
STATE_TRANSITION	re51	s29	s55		s29 * k1	Protein degradation
TRANSLATION	re52	s56	s29		s56 * k1	Protein translation
