# Supplementary material for: Dimerization and thiol sensitivity of the salicylic acid binding thimet oligopeptidases TOP1 and TOP2 define their functions in redox-sensitive cellular pathways
Source: Front Plant Sci. 2015 May 18;6:327. doi: 10.3389/fpls.2015.00327 (PMC4434903; doi:10.3389/fpls.2015.00327)
Supplement: Table S3 — Model parameters. [file Table3.PDF]

Table S3: Model parameters

Reaction ID	K constants	Value	Units
local:Reaction(re1)	k1	0.8	s^{-1}
local:Reaction(re5)	k1	0.5	s^{-1}
local:Reaction(re5)	k2	1	μM
local:Reaction(re8)	k1	0.5	s^{-1}
local:Reaction(re8)	k2	2	μM
local:Reaction(re12)	k1	0.1	s^{-1}
local:Reaction(re12)	k2	1	μM
local:Reaction(re13)	k1	0.1	s^{-1}
local:Reaction(re13)	k2	1	μM
local:Reaction(re15)	k1	0.05	s^{-1}
local:Reaction(re15)	k2	1	μM
local:Reaction(re16)	k1	0.05	s^{-1}
local:Reaction(re16)	k2	1	μM
local:Reaction(re19)	k1	0.008	s^{-1}
local:Reaction(re19)	k2	2	μM
local:Reaction(re20)	k1	0.005	s^{-1}
local:Reaction(re20)	k2	1	μM
local:Reaction(re22)	k1	0.5	s^{-1}
local:Reaction(re29)	k1	0.1	s^{-1}
local:Reaction(re29)	k2	1	μM
local:Reaction(re31)	k1	0.5	s^{-1}
local:Reaction(re31)	k2	2	μM
local:Reaction(re31)	k3	1	μM
local:Reaction(re31)	k4	0.2	μM
local:Reaction(re33)	k1	0.5	s^{-1}
local:Reaction(re33)	k2	1	μM
local:Reaction(re33)	k3	1	μM
local:Reaction(re37)	k1	0.1	s^{-1}
local:Reaction(re41)	k1	0.1	s^{-1}
local:Reaction(re41)	k2	1	μM
local:Reaction(re42)	k1	0.01	s^{-1}
local:Reaction(re42)	k2	10	μM
local:Reaction(re42)	k3	10	μM
local:Reaction(re43)	k1	0.2	s^{-1}
local:Reaction(re44)	k1	0.2	s^{-1}
local:Reaction(re46)	k1	2	s^{-1}
local:Reaction(re46)	k2	1	μM
local:Reaction(re47)	k1	1	s^{-1}

local:Reaction(re47)	k2	1	μM
local:Reaction(re48)	k1	0.005	s^{-1}
local:Reaction(re48)	k2	0.01	μM
local:Reaction(re49)	k1	0.005	s^{-1}
local:Reaction(re50)	k1	0.005	s^{-1}
local:Reaction(re51)	k1	0.001	s^{-1}
